# Urethral failure is not the predominant cause of female stress urinary incontinence

**DOI:** 10.1002/nau.25111

**Published:** 2022-12-12

**Authors:** Bo S. Bergström

**Affiliations:** ^1^ Department of Obstetrics & Gynecology Nordfjord Hospital Nordfjordeid Norway; ^2^ Department of Obstetrics & Gynecology Mora Hospital Mora Sweden

To the Editor

1

For many years, most stress urinary incontinence (SUI) theories have stated that urethral support failure is the predominant cause of SUI. This applies to the theories of Bonney, Ingelman‐Sundberg, Zacharin, Enhörning, Petros/Ulmsten, DeLancey, and Mostwin. Recently, DeLancey changed his opinion on the cause of SUI and now states that urethral function failure is the predominant cause.

In several articles, DeLancey et al. have argued for this change of opinion and claimed that correction of urethral function failure is necessary to avoid unsatisfactory treatment results.^1^, ^2^, ^3^, ^4^

This is unfortunate, as it may lead research on SUI in a wrong direction. The maximum urethral closure pressure at rest (MUCP) is irrelevant to the opening of the meatus internus and is not the predominant cause of SUI. MUCP is lower in women with SUI than in women with normal continence because it covaries with urethral support failure.^5^

Therapy intended to cure a presumed low urethral pressure will not cure SUI. The cure requires the prevention of bladder neck (BN) and proximal urethral funneling. McGuire, stated that the mid‐urethral area is not of particular interest in the assessment of passive continence and that closing pressure occurs anatomically at the BN.^6^, ^7^ Zacharin claimed that the urethra is normal in SUI^8^ and in a recent article Petros stated that urethral failure is not a critical factor in female urinary incontinence.^9^

DeLancey's change of opinion was due to the outcomes of a large prospective study on the relative importance of urethral support and urethral closure pressure, the ROSE study.^10^ This study was inadequately designed because it was based on an incorrect biomechanical model, the hammock theory (HT) of SUI. Consequently, the ROSE study is a failed study whose outcomes cannot be trusted. There is no hard data and strong evidence that urethral failure is the factor most responsible for SUI.^3^

The HT states that the intraabdominal pressure (Pabd) “transmission” to the proximal urethra is delayed during stress and that the urethra is pushed open when the bladder pressure exceeds the urethral pressure. These statements violate at least three laws of physics; consequently, HT is falsified and incorrect.^5^

The bladder and proximal urethra are inside a “water bag,” the abdominal cavity, and within a pressure equalization zone (PEZ), also called the abdominal pressure space, caudally limited by the pubocervical fascia (Figure 1). During stress, the generated pressure rise is equel and simultaneous in the entire PEZ, and consequently, the same in the bladder and proximal urethra (Pascal's law of hydrostatics). The expression “pressure transmission” is inappropriate because no pressure wave travels in any direction.

**Figure 1:**
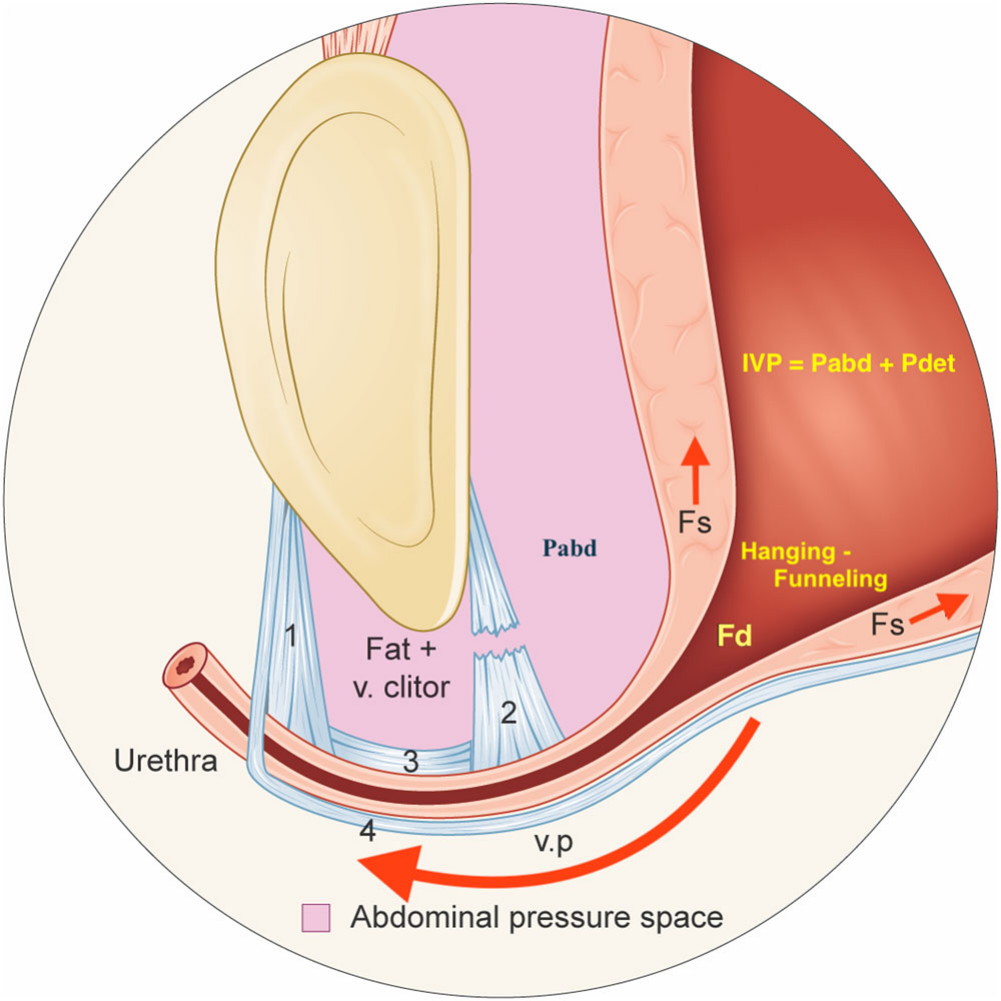
Illustration of hypermobile stress urinary incontinence during a Valsalva maneuver. In the illustrated case, the Pabd is just less than the abdominal leak point pressure (aLPP), and thus there is hanging/forced funneling without urine leakage. The maximum urethral pressure during stress (sMUP) resists the distending force (Fd) but the enforced distension of the proximal urethra may provoke urgency and frequency symptoms with or without uncontrolled detrusor contractions – explaining why most women with SUI have mixed symptoms. (1) Right anterior pubourethral ligament, which attaches to the pubocervical fascia (PCF), (2) right posterior pubourethral ligament which attaches to the PCF. The “fractured” PUL represents schematically a defected PUL not specifically a split PUL, (3) right intermediate pubourethral ligament, which attaches to the PCF (between this ligament and the os pubis, there is only fat and a ramus of vena clitoridis) and (4) PCF. Abdominal pressure space = pressure equalization zone. Fd, outflow distending force; Fs, pulling/shearing force; v. clitor, ramus of vena clitoridis; v.p., vaginal point (which corresponds to the attachment point of the posterior pubourethral ligaments [PUL] to the PCF on each side of the urethra), IVP, intravesical pressure; Pabd, intraabdominal pressure; Pdet, detrusor pressure. The illustration can alternatively be interpreted to demonstrate a urethra with minimal mobility (“fixed urethra”), exhibiting hanging/forced funneling, even at rest.

According to the urethral hanging theory (UHT) of SUI, the regularly measured decrease in urethral closure pressure during stress is caused by the hanging/forced funneling situation, which generates a pulling/shearing force (Fs) and an outflow distension force (Fd) that counterbalances/masks the fully (100%) transmitted Pabd. Pressure transmission to the proximal urethra is never delayed. The “delay” during stress—due to the unstable suburethral vaginal wall—corresponds to an equivalent “delay” in the entire PEZ.

Moreover, the HT statement that the meatus internus is pushed open is also incorrect because the bladder pressure is always perpendicular to the bladder wall (law of elastic collision) and does not generate a pulling force that can shear open/funnel the BN/proximal urethra. Additionally, a closed urethral lumen/meatus internus has an infinite resistance to urine flow (Hagen‐Poisseuille law). A pulling force is required to open the meatus internus.

Without a correct theory, knowing what to measure and how to interpret the results is impossible. This was clearly illustrated in the ROSE study.^10^ The authors measured 16 potential causal parameters for SUI but were still unsure if they had chosen the right support parameter for measurement.^3^ Therefore, they let an expert panel in a case‐control study further evaluate the ultrasound videos from the ROSE study regarding urethrovesical mobility during coughing.^11^ The experts blinded to the women's continence status could correctly identify women with SUI 57% of the time, which is only 7% better than expected by chance. The experts observed no consistent characteristic pattern of urethrovesical motion that could be correlated with stress incontinence. The authors concluded that the results confirm urethrovesical mobility is not strongly associated with stress incontinence. The authors meant that “since the experts could use all visible urethrovesical movements, if support were a major factor, it would have been noticed, and success with identification would be high.”^3^ This assumption is unlikely to be true.

According to UHT, the key support parameter for SUI is urethral support in relation to BN support (urethral mobility in relation to BN mobility). In hypermobile SUI, during stress, BN mobility is large and can be described as a dorsocaudal rotational movement. Initially, the BN and proximal urethra move synchronically; however, as the proximal urethra is less supported than the BN, it moves further until it breaks against (hangs on) the less mobile BN and is thereby deformed/funneled.

The difference in the rotational movement between the proximal urethra and BN is necessarily very small—they are tethered/interconected—just a few millimeters^12^, ^13^ and can hardly be detected by simply looking at a video, especially if the examiners do not know what to look for. It would be easier to detect the consequence of the small difference in mobility, that is, forced funneling of the BN/proximal urethra; however, this is difficult to detect. In a large 2005 study by Tunn et al., funneling was observed by ultrasound in 59% of women with SUI.^14^ This is despite the fact that 100% of SUI women indisputably must have funneling (i.e., opening of the meatus internus) during stress; otherwise, there would be no leakage of urine and no SUI.

In hypermobile SUI, funneling appears late in the dorsocaudal rotational movement. This is seen in a video produced by the Sydney Pelvic Floor Health Clinic at the University of Sydney, Australia.^15^ This video demonstrates how the Pabd presses down the suburethral vaginal wall and how the lower bladder is drawn/tented downwards until the more mobile proximal urethra breaks against the less mobile BN. In cases of minimally mobile BN, “fixed” type of SUI, hanging/forced funneling occurs even at rest (Figure 2).

The so‐called intrinsic sphincter deficiency (ISD) type of SUI, often defined as MUCP≤20 cm H2O or aLPP≤60 cm H2O and described as a form of SUI with weak sphincter and good support is in viewpoint of the UHT nothing less than a urethra that is hanging even at rest where the "weak sphincter" is the forced funneling of the proximal urethra and the “good support” is the urethra tethered to a minimally mobile BN, limiting its descent.

**Figure 2:**
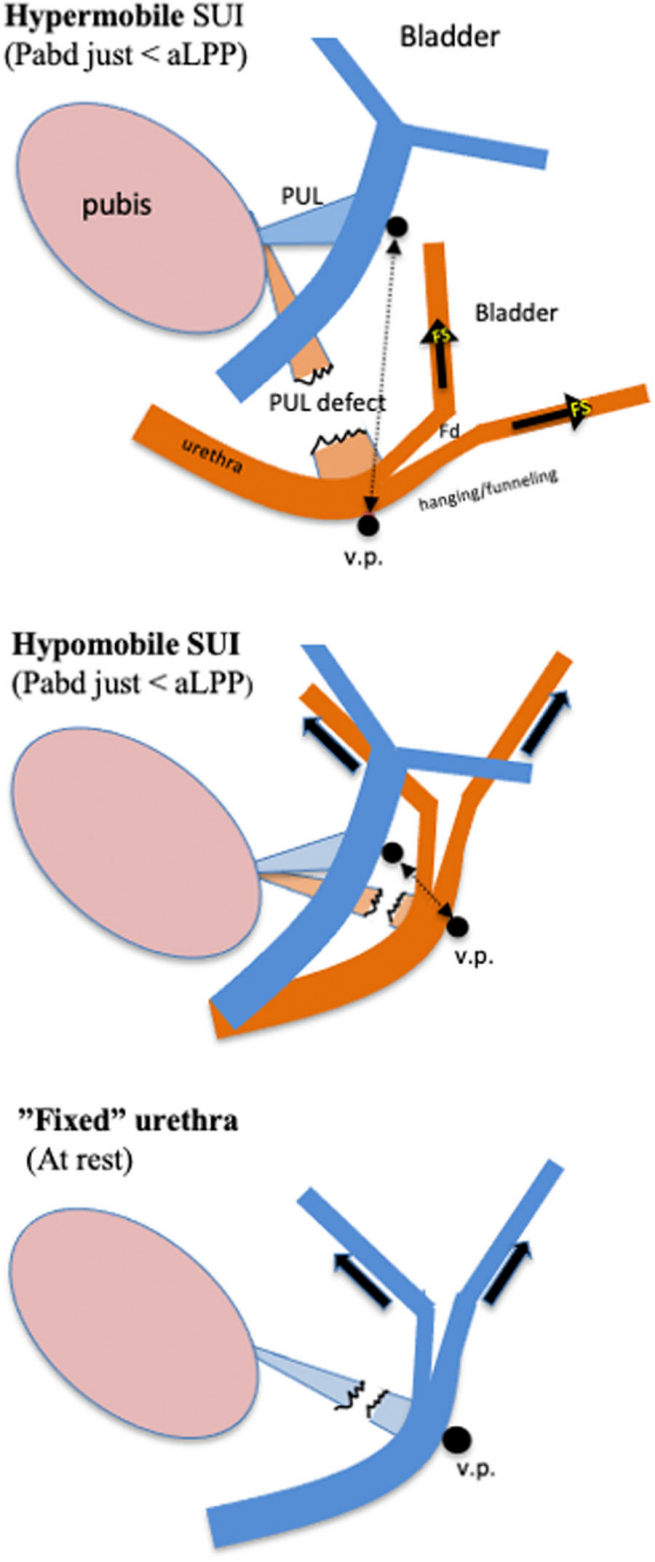
Demonstration of hanging/forced funneling in hypermobile, hypomobile and “fixed” types of SUI. It also shows the importance of the “therapeutic window” to choose between a tension‐free suburethral support and a lifting support. In cases with minimally mobile bladder neck (“fixed” urethra), a suburethral tension‐free tape is of marginal, if any, benefit to the woman. In these cases, the proximal urethra at the v.p. must be lifted above its resting position; it should be obvious that hanging/funneling existing even at rest cannot be corrected by an elastic tension‐free tape loosely placed under the posterior urethral wall. Lifting is also required in the cases with less hypomobile urethra not hanging at rest. This is because the use of tension‐free vaginal tape (TVT) or transobturator tape (TOT) is associated with low cure rates as the downward distance for the urethra to reach a hanging position is short, and a high Pabd makes the TVT and TOT sway downward a little owing to their elasticity. A TOT, in particular, sways downward because it is similar to a 5–8‐cm‐long horizontal hammock that is laterally fixed on soft tissues. This is in contrast to a TVT, which forms a tight vertical loop that is short because it adheres to the lower part of the bony pubic body postoperatively. To create a lift without the risk of obstruction, the “TVT technique” can be employed to insert one tuned tape in the paraurethral tissue on each side of the v.p. or alternatively to elevate the proximal urethra by broadly folding the pubocervical fascia at the v.p. and then supporting the plicated fascia with a TVT; the plicated fascia creates a broad cushion between the urethra and the tape that prevents obstruction problems. PUL, right posterior pubo‐urethral ligament which attaches to the PCF (the "fractured" PUL represents schematically a defected PUL and not specifically a split PUL); blue color, urethra at rest; brown color, urethra during stress; black arrow, therapeutic window (t.w.); Fs, pulling/shearing force; Fd, outflow distending force; aLPP, abdominal leak point pressure. The distance between the v.p. at rest and the v.p. at the abdominal leak point pressure is the “therapeutic window” (t.w.). An elastic tape remaining within the t.w. during stress is curative. The t.w. can be estimated by holding a fingertip a short distance under the v.p. at rest and asking the woman to perform a slow Valsalva maneuver. The maximum “curative” distance is the t.w. In hypermobile, hypomobile and “fixed” types of SUI, the t.w is large, small, and nearly zero, respectively.

The authors of the ROSE study were misled by the biomechanical model (HT) because the model mistakenly postulates that the meatus internus is pushed open when the bladder pressure exceeds the urethral pressure.^16^ Therefore, the authors had no indication to search for a support parameter, the failure of which could generate a pulling force that could shear open the meatus internus. Instead, several support parameters were indiscriminately chosen for measurement. The most predictive support parameter was point Aa corresponding to the urethrovesical junction/BN, which had an effect size of 0.5.^10^

If according to the UHT, the authors of the ROSE study had evaluated urethral support in relation to BN support  (urethral mobility in relation to BN mobility), they would have found an effect size much higher than 1.47 (MUCP) and concluded that urethral support failure is the most critical factor for SUI. BN mobility is irrelevant for SUI if the proximal urethral mobility is equal or lower. This is obvious in many women with a large urethrocystocele who are completely continent; however, if the cystocele and the BN are reduced without correcting for the proximal urethra descent, women will be incontinent because, during stress, the proximal urethra is pressed down and hangs on the BN (de novo SUI).

According to the UHT, the correct therapy for SUI is reinforcing the posterior pubourethral ligaments, thereby preventing the proximal urethra from descending to a hanging/forced funneling situation on a less mobile BN.

In hypermobile SUI, the tape should be set tension‐free with start 1 cm from the BN which implies that the center of the tape (width 11 mm) is positioned at the “vaginal point” (v.p)., that is, 15.5 mm from the BN. Accordingly, in case of a long urethra (45 mm), the tape position is proximal, and in the case of an average sized urethra (30 mm), the tape position is mid‐urethral.^5^ Conjecturally, a short urethra has a foreshortened extra‐abdominal part; consequently, the posterior PUL attachment to the vaginal wall may be found at approximately the same distance from the BN and equally at the midpoint of the intra‐PEZ urethra.

The elastic property of the polypropylen tape makes it to sway downward during stress implicating high failure rate for surgery in cases with a small therapeutic window, that is when the downward distance to urethral hanging is short (Figure 2). Therefore in the case of hypomobile or fixed‐type SUI, the proximal urethra at the v.p. should be lifted above its resting position before setting the tension‐free tape. A broadly folded suburethral fascia at the v.p. creates a lifting support and a broad cushion between the urethra and the tape, preventing obstruction problems.

It is given that a urethra which is hanging or almost hanging at rest cannot be treated by an elastic loosely placed, suburethral tape. To cure it is necessary to increase the margin to urethral hanging by a lifting support procedure.

Urethral function failure is not the predominant cause of SUI. The midurethral high‐pressure zone is irrelevant for opening the meatus internus. Urethral support failure generates the force that pulls open/funnels the BN and proximal urethra. Without forced funneling, there is no leakage of urine and no SUI, irrespective of low urethral pressure. MUCP is not a critical factor in SUI.

The efficacy of current treatments of SUI has plateaued – objective cure rate is 80% and subjective cure rate is 60% – not beacuse of untreated urethral function failure but because of mistreated urethral support failure. In the case of a long urethra, the tape is placed too distally, and in hypomobile or fixed type of SUI, the use of a tension‐free suburethral tape is unwarranted/ineffective, because the proximal urethra is not elevated above its resting position.^17^ A successful operation corrects urethral support failure and not urethral function failure.

## CONFLICT OF INTEREST

The author declares no conflict of interest.
